# Catalysis of amorpha-4,11-diene synthase unraveled and improved by mutability landscape guided engineering

**DOI:** 10.1038/s41598-018-28177-4

**Published:** 2018-07-02

**Authors:** Ingy I. Abdallah, Ronald van Merkerk, Esmée Klumpenaar, Wim J. Quax

**Affiliations:** Department of Chemical and Pharmaceutical Biology, Groningen Research Institute of Pharmacy, University of Groningen, 9713 AV Groningen, The Netherlands

## Abstract

Amorpha-4,11-diene synthase (ADS) cyclizes the substrate farnesyl pyrophosphate to produce amorpha-4,11-diene as a major product. This is considered the first committed and rate-limiting step in the biosynthesis of the antimalarial artemisinin. Here, we utilize a reported 3D model of ADS to perform mutability landscape guided enzyme engineering. A mutant library of 258 variants along sixteen active site residues was created then screened for catalytic activity and product profile. This allowed for identification of the role of some of these residues in the mechanism. R262 constrains the released pyrophosphate group along with magnesium ions. The aromatic residues (W271, Y519 and F525) stabilize the intermediate carbocations while T296, G400, G439 and L515 help with the 1,6- and 1,10-ring closures. Finally, W271 is suggested to act as active site base along with T399, which ensures regioselective deprotonation. The mutability landscape also helped determine variants with improved catalytic activity. H448A showed ~4 fold increase in catalytic efficiency and the double mutation T399S/H448A improved *k*_cat_ by 5 times. This variant can be used to enhance amorphadiene production and in turn artemisinin biosynthesis. Our findings provide the basis for the first step in improving industrial production of artemisinin and they open up possibilities for further engineering and understanding of ADS.

## Introduction

Amorpha-4,11-diene synthase (ADS) is an enzyme attracting world-wide interest as it is a sesquiterpene synthase that catalyzes the conversion of farnesyl pyrophosphate (FPP) to amorpha-4,11-diene, which is the precursor for the important antimalarial artemisinin. The first line treatment for malaria, as recommended by the World Health Organization (WHO), is Artemisinin-based Combination Therapy (ACT)^1,2^. Interestingly, this valuable antimalarial natural product in the past decade also gained massive attention for its anticancer properties^3–5^. Artemisinin is cytotoxic to cancer cells because its endoperoxide bridge interacts with iron producing free radicals that damage proteins and kill the cells^6,7^, which leads to an increasing demand for artemisinin and derivatives. Improved and sustainable methods for production including biosynthesis and semi-synthesis have been developed to overcome the problem of its low natural production^6,8^. ADS catalyzes the first and rate-limiting step in artemisinin biosynthesis^7,9^. Hence, it is important to study the structure of ADS to shed light on the involvement of the active site residues in the catalytic mechanism and create ADS variants with higher catalytic activity compared to the wild type.

Enzyme engineering to alter product specificity, thermostability and catalytic efficiency has been reported for a variety of classes of enzymes including terpene synthases. A thermostable mutant of 5-epi-aristolochene synthase has been reported^10^. Mutagenesis of the plasticity residues of *γ*-humulene synthase led to the generation of seven active synthases that produce different products^11^. As for amorphadiene synthase, the *ads* gene has been characterized^12^ and the enzymatic properties of ADS have been investigated^13,14^. In addition, a 3D model of ADS based on the 80% identical *α*-bisabolol synthase was created. The position of the co-factor magnesium ions and FPP substrate in the active site was established and verified^15^. The generation of mutability landscapes where a large number of protein variants are screened to discover the effect of each single amino acid substitution on enzyme activity, stability, and/or, selectivity is a valuable tool of protein engineering^16^. These landscapes produce detailed maps of favorable, neutral, and detrimental amino acids for each residue position in relation to different enzyme properties. This offers vital information on sequence-function relationships by highlighting functionally important residues and hotspot positions important for catalysis^16–19^. In this paper, we selected sixteen active site residues to create a mutability landscape of ADS. This mutability landscape is screened for catalytic activity and product profile to determine the residues involved in the mechanism of ADS and to select variants with enhanced catalytic activity compared to the wild type with the aim of improving the overall production of artemisinin. Finally, as a proof of concept, in *E*. *coli* the amount of amorphadiene produced by the highly active variants was compared to that of the wild type ADS.

## Results

### Selection of residues for site-saturation mutagenesis

The ADS model previously reported^15^ was used to examine active site residues and determine the best candidates for mutation. Residues within 5 Å radius of the FPP substrate in the active site were considered followed by computational examination of their location in relation to surrounding active site residues and predicted protein-substrate interactions. Based on further examination of reports on other enzymes of the terpene synthases family, residues corresponding to the metal ion binding motifs were disregarded, as their mutation usually results in loss of activity. In addition, the type of interactions between the residues and the substrate, bond distances and literature reports about the significance of corresponding residues in other terpene synthases were taken in consideration. It has been reported that aromatic residues play an important role in stabilization of the carbocation intermediates. Also, histidines are candidates to be the catalytic base in the active site essential for deprotonation and reprotonation while arginines were reported to participate in constraining the released pyrophosphate. Based on all that information, sixteen residues were selected for mutation namely, R262, W271, T296, H392, V396, T399, G400, G439, R440, H448, K449, L515, Q518, Y519, D523, and F525.

### Creation of ADS mutant library

**Figure 1:**
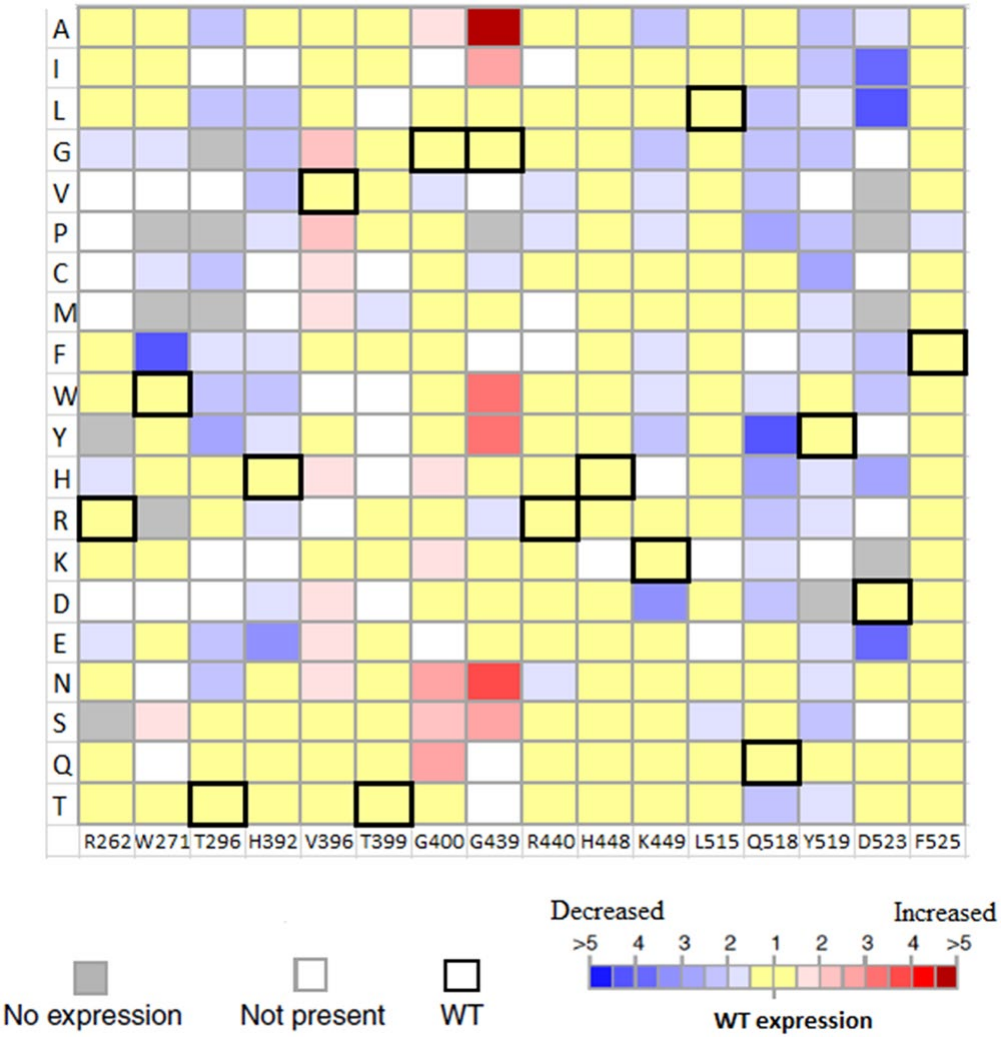
Mutability landscape of ADS for expression level using quantitative densitometric assay. For the mutability landscape, the vertical axis portrays the 20 possible amino acid residues. The wild-type amino acid residue at each position is indicated by bold squares, white squares represent variants that are not present in the library and grey squares represent variants that are not expressed. The color represents the concentration of expressed protein where blue range squares indicate decrease in expression while red range squares indicate increase in expression compared to the wild type.

To create the mutability landscape of ADS, an assortment of genes encoding possible variants of the selected residues was constructed. The typical use of NNN or NNS(K) degenerate codons during site saturation mutagenesis leads to codon redundancy, amino acid biases and generation of stop codons which decrease the quality of the library^20^. Hence, the strategy that uses a set of four degenerate codons; NDT, VMA, ATG, and TGG excluding the rare codons of *E*. *coli* and avoiding amino acid biases, was applied. Compared to the usual NNN or NNS(K), sequencing of 50 colonies per mutated residue produced a library with no rare or stop codons and with all amino acids represented equally. 95% of the sequenced mutants showed no deletions or undesired mutations. Approximately 85% of the desired mutants were obtained and all chemical groups of amino acids where present in the library. The missing mutants do not have significant impact on the quality of the library. This method is easy and fast where it allowed the generation and sequencing of a high quality library within one to two weeks^21,22^. A library in the total size of 258 variants was created. Finally, the library was purified and the concentrations of the variants were determined from the digital images of stained SDS gels (Fig. S1) using quantitative densitometric assay^17^. The landscape in Fig. 1 shows the expression levels of the different variants in the Library compared to the wild type.

### Mutability landscape of ADS for catalytic activity

**Figure 2:**
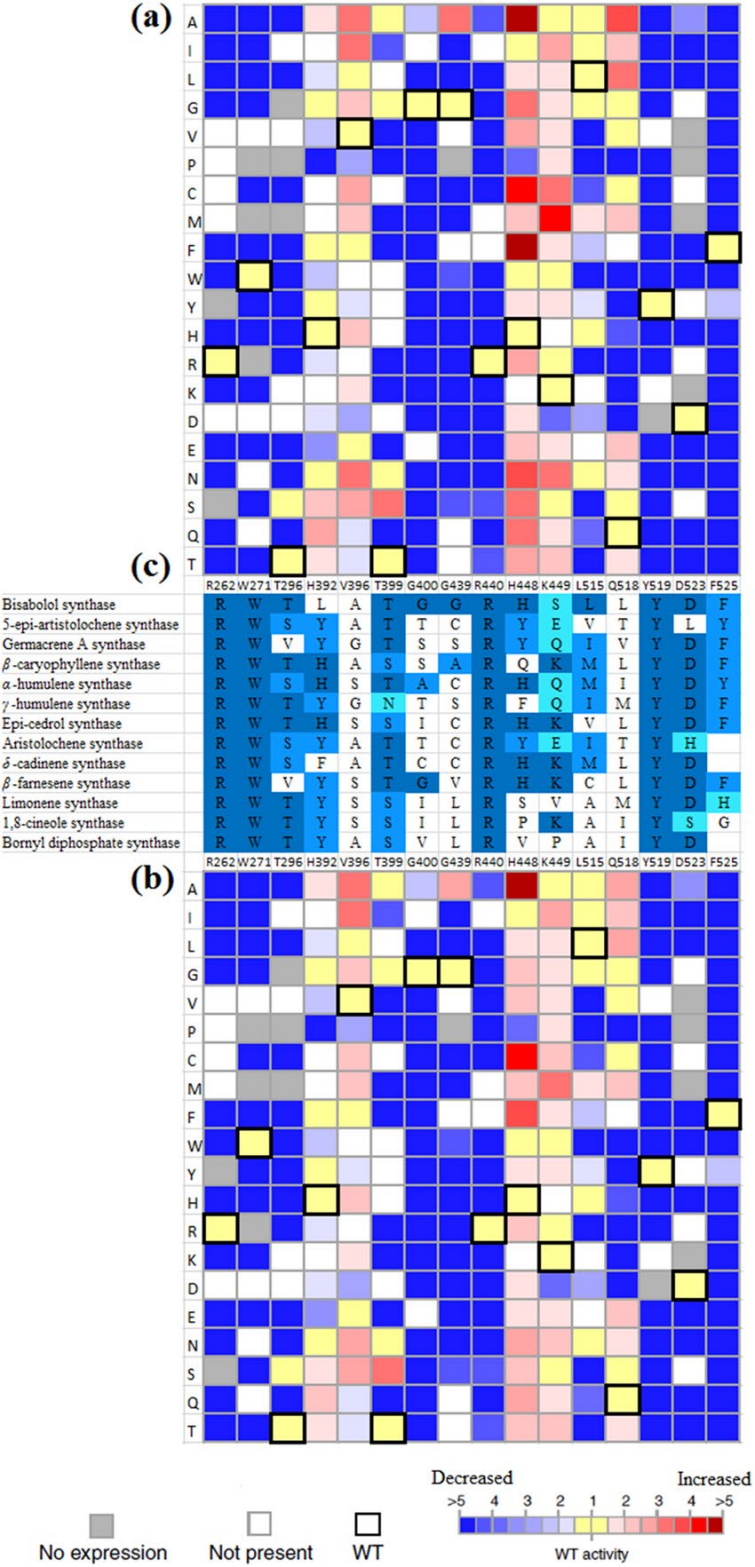
(**a**) Mutability landscape of ADS for catalytic activity using bioluminescent assay. (**b**) Mutability landscape of ADS for catalytic activity using GC-MS assay. For the mutability landscapes, the vertical axis portrays the 20 possible amino acid residues. The wild-type amino acid residue at each position is indicated by bold squares, white squares represent variants that are not present in the library and grey squares represent variants that are not expressed. The color represents the catalytic rate of reaction (V) where blue range squares indicate decrease in catalytic activity while red range squares indicate increase in catalytic activity compared to the wild type. The illustrated data are an average of two separate experiments (n = 2). (**c**) Multiple sequence alignment of the mutability landscape of ADS with ten sesquiterpene synthases and three monoterpene synthases. Residues that are identical, strongly similar, weakly similar and non-matching compared to ADS are colored dark blue, blue, cyan and white, respectively.

A mutability landscape for the catalytic activity of ADS was generated by examining the effect of each mutation on the production of amorpha-4,11-diene using two different assays. ADS converts the substrate FPP to amorpha-4,11-diene releasing inorganic pyrophosphate (PPi) in the process. The first assay depends on the fact that the amount of pyrophosphate released is equivalent to the amount of amorpha-4,11-diene produced. A rapid bioluminescent assay was used to measure the amount of pyrophosphate released continuously during the ADS reaction with FPP (Fig. S2), which is expressed as Relative Light Units (RLUs). The amount of light produced is directly proportional to the amount of PPi released during the reaction^23^. A curve of RLUs versus time is produced for all variants along with wild type ADS using the same concentration of protein and FPP (Fig. S3), where the slope of the linear part represents catalytic rate of reaction (V) as RLUs/sec for each sample. Hence, a mutability landscape comparing the catalytic activity of the wild type to the variants was generated (Fig. 2a). Variants with the darkest blue color in the landscape showed complete loss of enzyme activity whereas variants showing shades of red color indicated increase of activity compared to the wild type. The second assay was performed using GC-MS. Similar to the bioluminescent assay, a curve of amorphadiene peak area versus time is produced for each sample (Fig. S4), where the slope of the linear part represents the catalytic rate of reaction (V) as amorphadiene peak area/sec for each sample. Consequently, a mutability landscape based on the GC-MS assay was generated and compared to that produced from the bioluminescent assay (Fig. 2b).

The mutability landscapes showed that most variants of residues R262, W271, T296, G400, G439, R440, Y519, D523 and F525 completely lost their activity indicating that these residues play an essential role in ADS mechanism, which will be further explored in the 3D model to designate their function. In addition, some variants of residues H392, V396, T399, H448, K449 and Q518 demonstrated catalytic activity higher than the wild type. The position with the highest impact on catalytic activity is H448 where 15 of the variants improved the catalytic activity. The next step in screening the library would be examining the product profile compared to the wild type and linking it to the catalytic activity.

### Evaluation of the product profile of the ADS library using GC-MS

Following the screening of the library for catalytic activity, the GC-MS product profile of the library was compared to that of wild type ADS. Wild type ADS produces amorpha-4,11-diene as major product along with other sesquiterpenes as minor products as shown in its GC chromatogram (Fig. S5). The GC chromatograms of the variants in the library were compared to that of the wild type. Inactive variants showed no products at all in their GC chromatograms (Fig. S6) confirming their inactivity in the mutability landscape for catalytic activity. Residue L515 was found essential for product specificity of ADS as its mutation changed the product profile by decreasing the amount of amorphadiene produced and increasing the amount of *γ*-humulene or *α*-bisabolol. In addition, T296L and T296Y were found to produce the acyclic *β*-farnesene suggesting that they are unable to perform the cyclization step in the mechanism, similar as reported before for T296I and T296V^24^.

### Role of selected active site residues in the mechanism of ADS

**Figure 3:**
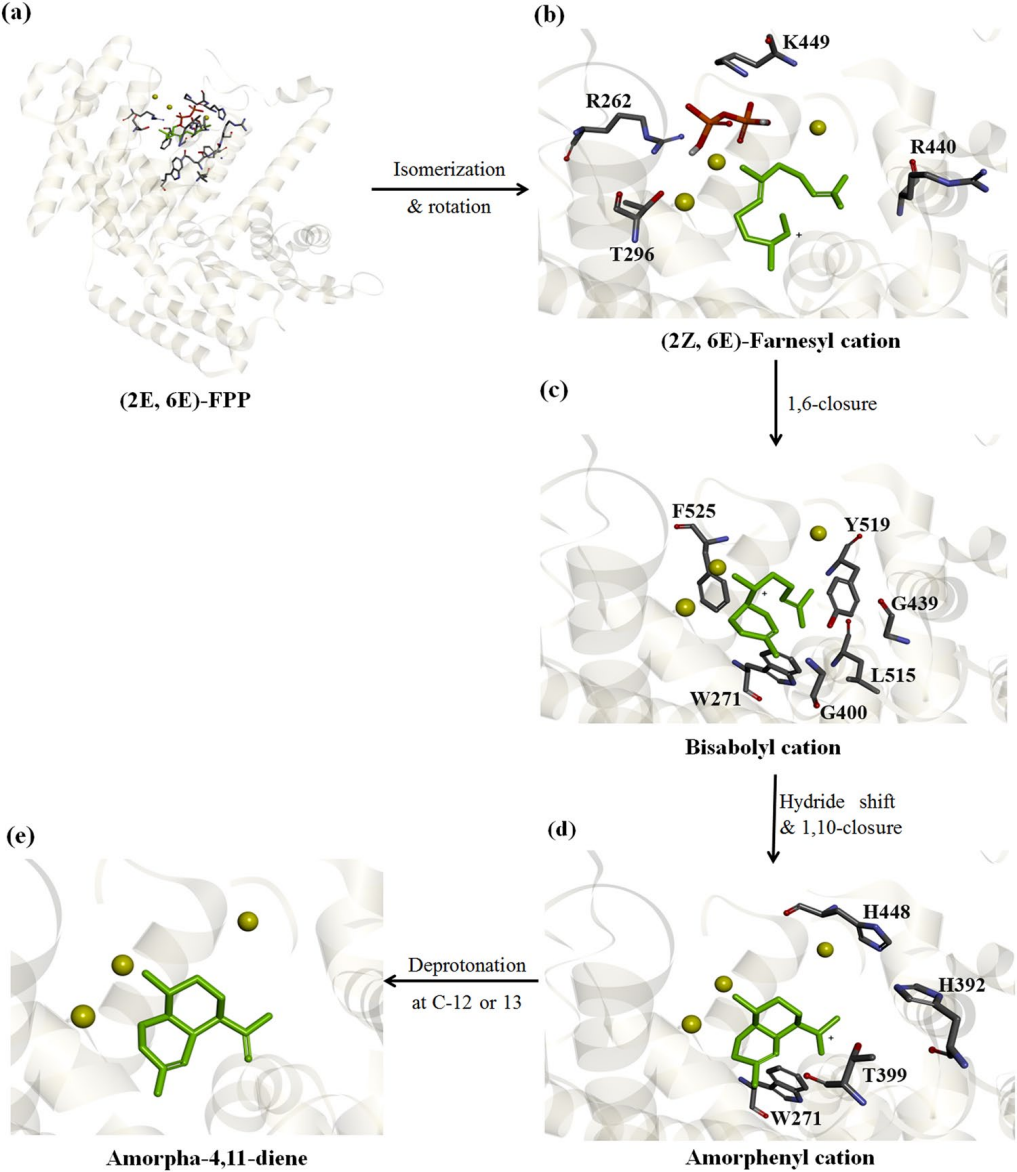
Proposed mechanism of ADS. (**a**) The substrate (2*E*, 6*E*)-FPP in the active site surrounded by the sixteen residues selected for mutation. (**b**) Following isomerization, the intermediate (2*Z*, 6*E*)-Farnesyl cation (green) and the released pyrophosphate (red) are in the active site. (**c**) 1,6-ring closure leads to the formation of the bisabolyl cation. (**d**) 1,10-ring closure produces the amorphenyl cation. (**e**) Deprotonation at C-12 or 13 creates the final product amorpha-4,11-diene.

ADS is a cisoid sesquiterpene synthase where its mechanism (Fig. 3) begins with an isomerization step at the Δ^2,3^ double bond of (2*E*,6*E*)-FPP followed by ionization to produce (2*Z*,6*E*)-farnesyl cation. The farnesyl cation undergoes 1,6-ring closure to give a bisabolyl cation. The next step is hydride shift in the bisabolyl cation and 1,10-cyclization to create the amorphenyl cation. Deprotonation at C-12 or 13 of the amorphenyl cation leads to the production of amorpha-4,11-diene as the major product. ADS can also produce minor amounts of other sesquiterpenes from side reactions along the pathway. Based on the mutability landscape of ADS for catalytic activity and the screening of product profile of the library, the involvement of certain residues can be deduced. Hence, residues that are essential for the mechanism were determined and their role explained as follows.

The sequence of ADS was aligned with several monoterpene and sesquiterpene synthases (Figs 2c and S8) to spot the similarities and differences between them to provide a picture about the involvement of different residues in the mechanism. Among these sesquiterpene synthases are *α*-bisabolol synthase, 5-epi-aristolochene synthase, germacrene A synthase, *β*-cayophyllene synthase, *α*-humulene synthase, *γ*-humulene synthase, epi-cedrol synthase, aristolochene synthase, *δ*-cadinene synthase and *β*-farnesene synthase along with the monoterpene synthases: limonene synthase, 1,8-cineole synthase and bornyl diphosphate synthase. *β*-farnesene synthase lacks cyclization and produces the acyclic sesquiterpene *β*-farnesene. *α*-bisabolol synthase performs only 1,6-cyclization to create the monocyclic *α*-bisabolol while germacrene A synthase just does 1,10-ring closure to generate the monocyclic germacrene A. Epi-cedrol synthase is another sesquiterpene synthase from *Artemisia annua* like ADS. The enzymes *α*-humulene and *γ*-humulene synthase execute 1,11-cyclization to produce their major product. *δ*-cadinene synthase is a cisoid sesquiterpenes synthase as ADS but with slightly different ring cyclizations. Aristolochene synthase is also a cisoid sesquiterpene synthase that performs an initial 1,10-ring cyclization followed by 1–6, ring closure. 5-epi-aristolochene synthase is a transoid sesquiterpene synthase that does not carry out the initial isomerization step and produces 5-epi-aristolochene as its major product^25–29^. Figure 2c shows, among the sixteen residues in the mutability landscape, those that are identical, strongly similar, weakly similar and non-matching compared to ADS. The position of the sixteen residues in the active site is highlighted in Fig. 3a.

#### Residues involved in constraining the released pyrophosphate in the active site

The arginine and lysine residues (R262, R440 and K449) located in the active site of ADS within 4 Å of the substrate are present in the protonated form carrying a positive charge so they cannot act as the active site base. However, they can play a role in constraining the PPi moiety released from the substrate (Fig. 3b), where these residues along with the magnesium ions may bind the negatively charged PPi group to prevent it from recapturing the formed reactive carbocations^9,30,31^. ADS variants of R262 including R262K showed loss of catalytic activity indicating its important role in restricting the PPi group. It is noteworthy to mention that due to the guanidinium group of arginine, it can perform a larger number of interactions compared to lysine^32^. Hence, the R262K variant was inactive. It was hypothesized that R264 and R441 of 5-epi-aristolochene synthase (5-EAS) perform this role^30^. Thus, it was suggested that R262 and R440 in ADS corresponding to those in 5-EAS would act similarly^9^. However, R440A, R440S and R440T show very low activity, in addition, the side chain of R440 is pointing away from the active site with more than 10 Å bond distance separating it from the PPi. The situation is similar with the R440 in the crystal structure of *α*-bisabolol synthase (BOS). By examining the crystal structure of 5-EAS, it is clear that the side chain of R441 is directed towards the active site unlike ADS and BOS but the bond distance was still too big to allow for interaction with the PPi. Finally, K449 variants retained their activity so it does not seem to be involved with this function. It can be concluded that only R262, along with the magnesium ions, are responsible for binding the PPi group and directing it away from the carbocations in the active pocket. Furthermore, R262 is conserved in several monoterpene and sesquiterpene synthases (Fig. 2c). Also, R440 is conserved which might indicate that it has another role in the mechanism but not this one.

#### Aromatic residues play an essential role in the mechanism

The vital role of aromatic residues in stabilizing the carbocation intermediates in the active site of sesquiterpene synthases is discussed in literature^15,31,33,34^. Amid the aromatic residues lining the active site of ADS, three residues (W271, Y519 and F525) are within 3 Å radius of the substrate with their phenyl rings angled towards the active site showing π-alkyl interactions with the substrate (Fig. 3c). By examining the mutability landscape of ADS, all variants of these residues showed no catalytic activity in the bioluminescent assay and no products in their GC-MS chromatograms indicating loss of enzyme activity except for F525Y which retained low activity. This proves the essential role of these aromatic residues in the mechanism where they can stabilize the intermediate carbocations until production of amorpha-4,11-diene. In addition, sequence alignment of ADS along with several sesquiterpene and monoterpene synthases (Fig. 2c) showed that these aromatic residues are conserved.

#### Residues involved in 1,6- and/or 1,10 ring closure

ADS mechanism involves two ring closures, first is 1,6-closure to produce bisabolyl cation followed by 1,10-closure to produce amorphenyl cation. It has been reported that T296V lost its cyclization ability producing the acyclic *β*-farnesene instead of amorpha-4,11-diene^24,35^. T296L and T296Y showed the same pattern of behavior. It is deduced that T296 is involved in the 1,6-ring closure (Fig. 3b). The variant T296S retains the cyclization ability suggesting that the hydroxyl group is involved. In addition, this residue is conserved as threonine or serine in several terpene synthases except for *β*-farnesene synthase which produces an acyclic product and germacrene A synthase which lacks 1,6-ring closure. However, T296A also retains the cyclization ability which indicates that the hydroxyl group is not necessary. As for 1,10-ring closure, variants G400A, G439A, L515A, L515G, L515C, L515S and L515T accumulated more *α*-bisabolol compared to the wild type indicating impaired 1,10-cyclization. This suggests that residues G400, G439 and L515 play a role in the success of the 1,10-ring closure.

#### Determination of the active site base of ADS

The active sites of terpene synthases must possess a base that mediates the series of deprotonations and reprotonations including the final deprotonation to yield the major product. In the ADS model, H392 and H448 are two apparent basic residues in the active site (Fig. 3d). The imidazole side chain of these histidines can act as the functional active site base. However, the positioning of these residues with respect to the substrate in the model proves difficult to perform the deprotonations. In addition, most variants of H392 and H448 retained their catalytic activity as proven by the mutability landscape of ADS (Fig. 2a and b). Hence, H392 and H448 can be excluded as the active site base. Researching through literature, a less orthodox possibility arises where a tryptophan residue (W273) has been previously reported as the active site base of 5-epi-aristolochene synthase^30,31^. Within the active site of ADS, W271 corresponds to W273 of 5-EAS. Thus, we suggest that W271 can perform the same role in ADS and act as the active site base. As shown in Fig. 3d, the final carbocation possesses a positive charge at C11 which increases the acidity of the proton at C12 that is directed towards W271. This proton can now be removed by W271 producing a positive arenium ion (WH^+^) and the final major product amorpha-4,11-diene. The fact that W271 can act as the active site base is corroborated by the observed loss of activity of all variants, aromatic or non-aromatic, of this residue similar to 5-EAS and the tryptophan-271 residue is conserved in several monoterpene and sesquiterpene synthases as shown in Fig. 2c.

#### Residues responsible for regioselective deprotonation to produce amorpha-4,11-diene

Residue T399 was reported to be responsible for regioselective deprotonation to produce amorpha-4,11-diene as the major product instead of amorpha-4,7-diene^35,36^. This was confirmed by loss of activity of T399P, T399M, T399F, T399R, T399K, T399E and T399Q variants or the increase in the level of amorpha-4,7-diene by T399A, T399I, T399G, T399V and T399N variants indicating loss of regioselective deprotonation. The only variant that retained the production of amorpha-4,11-diene as the major product is T399S indicating the involvement of the hydroxyl group in this process, which is further substantiated by the conservation of this residue as threonine or serine in several terpene synthases (Fig. 2c).

### Enhanced catalytic activity of ADS

In the mutability landscapes of ADS, comparison of V (RLUs/sec or amorphadiene peak area/sec) of each variant with the wild type showed hot spots with improved catalytic activity (Fig. 2a and b). In addition, the product profile of such variants was evaluated to ensure it is similar to that of the wild type (Fig. S7). Some variants of residues Q518, H392, H448, K449, V396, T399 and G439 showed up to five fold increase in catalytic activity compared to wild type. Position H448 had the most impact on catalytic activity where 15 of the produced variants showed different degrees of improved activity compared to the wild type. H448A displayed the highest increase in catalytic activity where it showed, in both bioluminescent and GC-MS assays, around 5-fold increase in catalytic rate of reaction. Variants H448F and H448C displayed 4-fold increase in activity, H448N, H448S and H448Q exhibited approximately 3-fold increase while H448G, H448V, H448M, H448R and H448T showed around 2-fold increase. Also, variants H392A, H392I, H392N, V396A, V396I, G439A, K449M, Q518A and Q518L displayed between 3 to 4-fold increase in catalytic activity compared to wild type. To further improve the catalytic activity of ADS, double mutants of the variants with improved activity compared to wild type were created. Four double mutants namely; T399S/H448A, T399S/H448F, T399S/V396A and T399S/V396I were constructed. These double mutants were screened by the bioluminescent assay to determine their catalytic activity along with the wild type and corresponding single mutants. However, the double mutants showed V close to the single mutants H448A, H448F, V396A and V396I, respectively with the variant T399S/H448A slightly better than the single mutants. Furthermore, kinetic measurements of K_m_ and *k*_cat_ of the wild type, H448A, T399S and T399S/H448A were performed using GC-MS to determine the catalytic efficiency (Table 1). The variant H448A showed a *k*_cat_ 3.5 times higher than the wild type with their K_m_ nearly the same. The catalytic efficiency (*k*_cat_/K_m_) of H448A was improved by ~4 folds compared to the wild type. The previously reported variant T399S showed similar results of nearly 2 fold increase in *k*_cat_^35,36^. Finally, the double mutant T399S/H448A improved the *k*_cat_ by 5 folds compared to wild type, but at the expense of a raised K_m_ making its catalytic efficiency ~3 times higher.

**Table 1:** Steady state kinetic parameters of wild type ADS and selected variants with FPP as the substrate.

Enzyme	*k*_cat_ (s^−1^)	K_m_ (μM)	*k*_cat_/K_m_ (s^−1^.M^−1^)
ADS^WT^	0.20 ± 0.007	5.47 ± 0.558	3.6 × 10^4^
ADS^H448A^	0.68 ± 0.03	5.04 ± 0.753	13.5 × 10^4^
ADS^T399S^	0.39 ± 0.06	5.89 ± 1.278	6.6 × 10^4^
ADS^T399S/H448A^	1.00 ± 0.14	8.50 ± 1.605	11.8 × 10^4^

### Comparison of *in vivo* amorphadiene production levels of highly active variants and wild type

**Figure 4:**
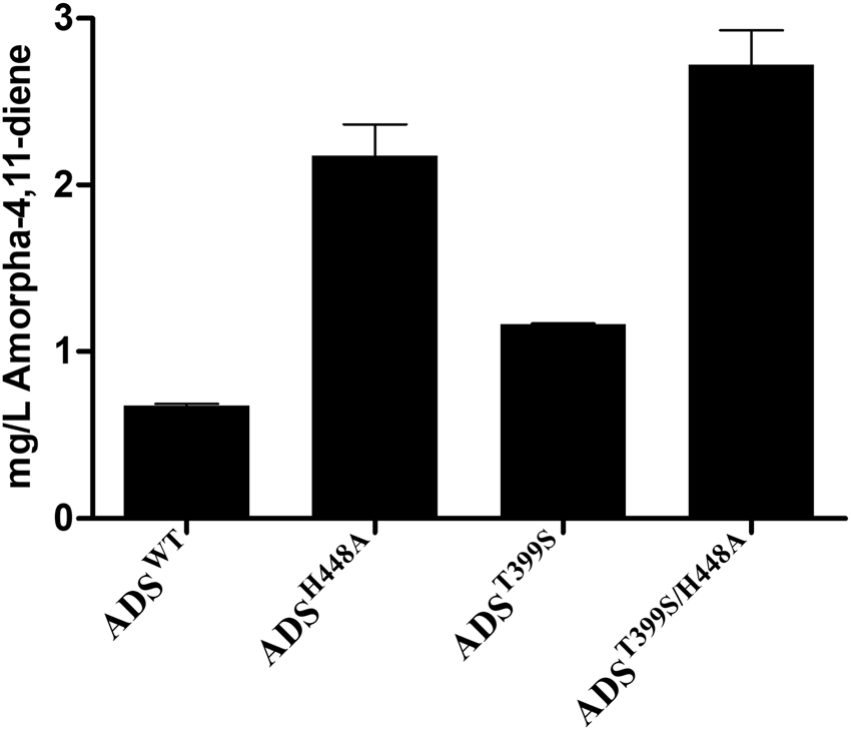
Comparison of *in vivo* production levels of amorpha-4,11-diene in *E*. *coli* due to expression of wild type ADS and variants H448A, T399S and T399S/H448A. The illustrated data are an average of two separate experiments (n = 2).

*E*. *coli* was used as a cell factory to compare the amount of amorpha-4,11-diene produced *in vivo* by expression of wild type ADS, H448A, T399S and T399S/H448A. These three variants, with higher *K*_cat_, showed increased production levels of amorpha-4,11-diene compared to the wild type (Fig. 4). H448A (2.2 mg/L) and T399S (1.2 mg/L) simultaneously showed ~3 and 1.7 times higher amorphadiene production compared to the wild type (0.7 mg/L). The double mutant T399S/H448A (2.7 mg/L) caused ~4 fold increase in the amount of amorphadiene produced. The higher amorphadiene production by these three variants corroborates that the *K*_cat_ of ADS is rate limiting in the synthesis of artemisinin.

## Discussion

With the aid of a reliable 3D model of ADS^15^ and the knowledge of the structure of its active site, a rational design of a mutability landscape of ADS has been performed. A mutant library of the sixteen active site residues was generated showing high diversity of amino acids and their locations in the active site. With this library we have identified residues involved in the catalytic mechanism and obtained variants with improved activity compared to the wild type. A fast, reliable assay for screening the catalytic activity of the mutant library was indispensable and GC-MS, the most widely used assay for the detection of volatile terpenoids and evaluation of the kinetic efficiency, was deployed by us for high throughput screening^36,37^. In addition we have tested the catalytic activity by measuring the amount of released PPi using the theory of a recently described colorimetric malachite green assay, which was confirmed to be comparable to the GC-MS and radioactive assays^38^. The bioluminescent assay has been used by us in a 96-well format allowing for rapid screening of the library. The challenge was to determine a good ratio between protein and FPP concentration to produce a luminescence signal that can be accurately detected by the plate reader. ADS concentration of 0.15 μM in combination with 50 μM FPP was found to be optimal. The high amount of FPP served to ensure that its concentration is above the K_m_ of the enzyme so the assay would give an approximate indication about the *k*_cat_ of the different variants. This assay was complementary to the GC-MS assay that allowed screening the whole library for product profile and selected variants for catalytic activity using a time point assay.

In summary, screening of the mutability landscape proved that R262 creates a region of high positive charge along with the magnesium ions in the active site to bind and neutralize the negative charge of the released PPi preventing it from interfering with the reactive carbocations. Evidence showed that R440 and K449 were not involved. The importance of aromatic residues (W271, Y519 and F525) for stabilization of the reactive carbocations in the active site was confirmed. Residues involved in 1,6- or 1,10-ring closure were suggested. H392 and H448 were excluded as the active site catalytic base while W271 was suggested as a possible candidate for this role. Another possibility would be that a water molecule that is present in the close vicinity of the amorphenyl cation and W271 can act as the active site base. This will also lead to the competitive production of the alcohol amorpha-4-en-7(11)-ol^35^. A supplementary video illustration of the mechanism of ADS is provided. Finally, several variants having the same product profile as wild type ADS showed improved catalytic activity especially variants of residue H448. Mutation of H448 may have improved the release of the product from the active site or contributed to the stabilization of the carbocations due to its location near the active site opening and its interactions with the magnesiums, pyrophosphate and surrounding residues. This provided excellent candidates for mutants with higher catalytic efficiency than the wild type especially H448A, which showed ~4 fold increase in catalytic efficiency. Furthermore, T399S/H448A increased *k*_cat_ by 5 times compared to wild type, but also raised the k_m_. These variants are better than the reported T399S and T447S that only show 2-fold higher turnover rate (*k*_cat_). T399S/H448A variant is also better thanT399S/T447S that only tripled the *k*_cat_ but at the expense of higher K_m_ compared to wild type^35,36^. Hence, the double mutant T399S/H448A can be very instrumental in improving the production of amorpha-4,11-diene and in turn that of artemisinin in large scale industrial fermenters where a slightly raised K_m_ is not a hindrance as opposed to the advantage of the 5 fold increase in the turnover rate.

Commercial production of artemisinin suffers from fluctuation in price and availability of plants crops. This led to the semi-synthetic artemisinin project using yeast cells which was approved by the WHO and produced by Sanofi^5,6^. Since ADS catalyzes the first committed and rate-limiting step in artemisinin biosynthesis^7,9^, the up-regulation of ADS is considered a good strategy for improving the production of artemisinin. In the future, ADS variant T399S/H448A, with improved turnover rate (*k*_cat_), can be incorporated into the yeast cells to enhance the production of the amorphadiene with the goal of boosting the manufacturing of artemisinin. As a proof of concept, the amount of amorphadiene produced in *E*. *coli* due to expression of the variants and the wild type were compared. It can be seen that the higher the *k*_cat_ of the variant, the higher the amorphadiene production compared to the wild type. Hence, incorporating such variants into industrial microbial strains with improved flux of the upper metabolic pathway will show similar increase in amorphadiene production levels compared to wild type at higher titers. Also, the possibility of using these variants in transgenic *Artemisia annua* can be explored. The increased availability of artemisinin will provide a chance to lower the price and increase the supply to third world countries. In addition, it will open the door to explore applications of artemisinin beyond antimalarial, especially research in combining iron and artemisinin to target cancer cells.

## Methods

### Creation of ADS mutant library

Residues for mutation were selected by examining the active site of the reported ADS model using Discovery Studio 4.5 software (Accelrys, CA, USA). A full-length cDNA encoding ADS (GeneBank/NCBI accession number AY006482) in the pET15b vector in frame with the N-terminal histidine tag was used as the template^15^. The small-intelligent method^20^ was implemented for full randomization of each residue using a mixture of four pairs of complementary primers supplied by Eurofins with the degenerate codons NDT, VMA, ATG, and TGG at a ratio of 12:6:1:1, respectively. Quikchange method was applied to create the library where 50 μl PCR reactions with the primer mixture were performed using Phusion high fidelity DNA polymerase followed by *Dpn*I digestion and transformation into *E*. *coli* XL1-blue. The produced colonies per residue were sequenced by GATC Biotech. Each desired colony was separately grown in Luria−Bertani (LB) broth supplemented with 100 μg/ml ampicillin. All plasmids, each containing a single mutant ADS gene, were isolated from their corresponding cultures using the NucleoSpin 96 Plasmid Core Kit (Bioke´). Then these isolated plasmids were transformed individually into chemically competent *E*. *coli* BL21 (DE3). Each *E*. *coli* BL21 transformant retaining a pET15b vector with a unique ADS gene constituting the library was stored at −80 °C until further use.

### ADS library expression, purification and quantitation

The *E*. *coli* BL21 (DE3) variants were inoculated into 1 ml LB medium containing 100 μg/ml ampicillin and grown overnight at 37 °C. Overnight cultures were diluted to OD_600_ of 0.05 in 15 ml auto-induction medium [phosphate buffer (pH 7.2), 2% tryptone, 0.5% yeast extract, 1% NaCl, 0.6% glycerol, 0.05% glucose and 0.2% lactose] supplemented with 100 μg/ml ampicillin. The cultures were grown at 37 °C, 250 rpm until OD_600_ of 0.7. Then, incubated overnight at 20 °C, 190 rpm. Cells were collected by centrifugation, 10 min at 4000 rpm, 4 °C, followed by three cycles of freezing and thawing then resuspended in 1.5 ml lysis buffer (50 mM Tris-HCl, pH 7.5–8.0, 100 mM NaCl, 10 mM *β*-mercaptoethanol, cOmplete™ EDTA-free protease inhibitor cocktail tablet and 1 mg/ml lysozyme). Cells were lysed by incubation at 20 °C, 250 rpm for 30 min, and the soluble protein fractions were collected by centrifugation, 10 min at 4000 rpm. Proteins were then purified using His MultiTrap™ Fast Flow GE Healthcare 96-well plates using (20 mM Tris-HCl, 10 mM MgCl_2_, 150 mM NaCl, 1 mM DTT and 20 mM imidazole, pH 7.4–8) as binding and wash buffer, and elution buffer with 10% glycerol and 250 mM imidazole. To quantify the ADS concentration of each variant, 24 μl of each purified protein was boiled for 5 min. with 6 μl of protein sample buffer. The boiled samples (15 μl) were loaded on pre-cast 4–12% polyacrylamide gels (NuPAGE Novex) with MOPS buffer. Five wild type ADS samples (15 μl) with standard concentrations of 100, 250, 500, 750 and 1000 ng/μl were loaded on the gel along with the purified variants and served as calibration samples for quantification. The concentration of the standards was determined by the coomassie (Bradford) protein assay^39^. The gels were stained using the Coomassie-based stain InstantBlue (Expedeon Ltd) after electrophoresis (at 150V for 1 h). A digital image of the gel was snapped with the aid of Chemi Genius2 Bio Imaging System (Syngene, Cambridge, UK). The pictures of the SDS gels were used for the densitometric concentration assessment (Fig. S1). The concentrations of the ADS variants in the library were calculated based on their band size and intensity in the digital image of the gel, compared to the calibration samples by using the software GeneTools (version 4.02, Syngene). The standard calibration samples were loaded on each gel to quantify only the samples on that gel.

### Bioluminescent PPiLight™ inorganic pyrophosphate assay for ADS catalytic activity

Perkin Elmer JANUS 8-tip Varispan Automated Liquid Handling Workstation was used for high throughput screening of the library. This assay measures the amount of PPi released from the conversion of the FPP substrate to amorpha-4,11-diene by ADS. The released PPi converts AMP to ATP which in turn produces light through the enzymatic reaction of luciferase enzyme^23^. Reactions containing 0.15 μM ADS protein and 50 μM FPP in 10 mM MgCl_2_, 2 mM DTT, 10 mM Tris-HCl buffer (pH 7.4) were mixed with 20 μl Lonza PPiLight™ converting and detection reagent mixture to a final volume of 60 μl in opaque white wall flat bottom 96-well microtiter plates (Greiner LUMITRAC™ 600 microplates). The plates were place immediately in a preheated to 30 °C chamber of FLUOstar Omega Plate reader with moderate shaking and the luminescence signal was recorded continuously for 2 hrs and reported as Relative Light Units (RLUs). The catalytic rate of reaction (V) of each variant was presented as RLUs/sec and compared to wild type.

### GC-MS assay for ADS catalytic activity

Perkin Elmer JANUS 8-tip Varispan Automated Liquid Handling Workstation was used for high throughput screening of the library. Four 0.5 ml reactions of each variant containing purified enzymes (0.038 µM) in 10 mM Tris-HCl buffer (pH 7.4), containing 10 mM MgCl_2_, 2 mM DTT, and 10 μM FPP substrate were overlaid with 200 µl hexane containing tetradecane internal standard. The reactions were incubated at 30 °C then stopped by addition of an equal volume of 0.2 M KOH containing 0.1 M EDTA at 3, 6, 9 and 12 min, respectively. Two microliters of the n-hexane extracts were analyzed in total ion scan using a HP-5MS (5%-Phenyl)-methylpolysiloxane column (Agilent J&W 0.25 mm inner diameter, 0.25 µm thickness, 30 m length) on a Shimadzu GCMS-QP2010SE system equipped with a GC-2010 Plus high performance gas chromatograph. It was injected splitless onto the GC column using helium as the carrier gas. The injector temperature was 250 °C; the oven initial temperature was 50 °C with an increment of 5 °C/min up to 180 °C and then up to 300 °C with an increase of 10 °C/ min. The solvent cut-off was 5 minutes. The peak area of amorphadiene was determined in each sample. A curve of amorphadiene peak area versus time was generated for the wild type and the variants (Fig. S4). The catalytic rate of reaction (V) of each variant presented as amorphadiene peak area/sec was calculated and compared to wild type.

### GC-MS assay for ADS product profile

Similarly, an *in*-*vitro* GC-MS assay was conducted to determine the product profiles of the library. Purified enzymes (50 μg) was assayed in 0.5 mL reactions with 46 μM FPP substrate and incubated for 1 hour. The product profile of all samples was compared to that of reference wild type ADS. The products in the chromtaograms were identified by comparing their mass spectra to spectra included in the NIST (National Institute of Standards and Technology, Maryland, USA) and other libraries.

### GC-MS assay for measuring enzyme kinetics

Similarly, a fixed concentration of purified enzyme is used along with a range of FPP concentration from 0 to 50 µM. Six 0.5 ml reactions of each FPP concentration, consisting of purified enzyme (0.03 μM wild type ADS, 0.01 μM H448A, 0.02 μM T399S and 0.01 μM T399S/H448A) with respective FPP concentration were incubated. Reactions were stopped at 3, 5, 7, 10, 15 and 20 min, respectively. Using Shimadzu OPTIC multimode inlet, 100 µl of the n-hexane extracts were injected at 60 °C with gradient increase up to 300 °C and 25 sec. vent time onto a chromosorb liner then to the GC-MS system. The oven initial temperature was 50 °C with an increment of 20 °C/min up to 300 °C. *β*-caryophyllene was used as standard to determine the concentration of amorphadiene. The MS instrument was set to selected ion mode (SIM) for acquisition, monitoring *m*/*z* ion 189 for amorphadiene and *β*-caryophyllene. The chromatographic peak areas for amorphadiene and *β*-caryophyllene were determined using the integration tools in GCMSsolution 1.20 software (Shimadzu, Den Bosch, The Netherlands). A calibration curve of standard *β*-caryophyllene with concentration range of 0.09–10 μM was created. For quantification of amorphadiene, the peak area for each sample was corrected by using the peak corresponding to the internal standard tetradecane. The amorphadiene concentration in each sample was calculated by applying the linear regression equation resulting from the *β*-caryophyllene calibration curve to each adjusted amorphadiene peak area. The concentration of amorphadiene was represent as μM *β*-caryophyllene equivalent^40^.

### Production, extraction and quantification of amorpha-4,11-diene in *E*. *coli*

Overnight cultures of the strains with different variants were grown in 2xYT medium containing ampicillin. The following day, the overnight cultures were diluted to an OD_600_ of 0.07–0.1. The cultures were incubated for 3 h at 37 °C and 220 rpm. Then, induction was started by adding IPTG to a final concentration of 1 mM. The cultures were overlaid with 100 μl dodecane containing 10 μl of 700 μM *β*-caryophyllene as internal standard. Then the cultures were grown overnight at 20 °C and 220 rpm. The next day extraction was performed by adding 200 μl hexane to the cultures overlaid with dodecane, followed by centrifugation for 10 min at 11000 rpm to separate the aqueous and organic phases. The dodecane-hexane layer was extracted for GC-MS analysis. The dodecane-hexane extracts were analyzed on an HP-5MS (5% Phenyl)-methylpolysiloxane column (Agilent J&W 0.25 mm inner diameter, 0.25 μm thickness, 30 m length) in a Shimadzu GCMS-QP2010SE system equipped with a 17A gas chromatograph (GC) and AOC-20i autoinjector. The extracts (2 μL) were injected splitless onto the GC column. The injector temperature was 250 °C, and the oven initial temperature was 100 °C with an increase of 10 °C per minute up to 210 °C. After 210 °C was reached the temperature was raised to 280 °C with an increase of 35 °C per minute and held for 2 min. The solvent cutoff was 4 min. The amount of amorphadiene was quantified as previously mentioned in literature^40^.

## Electronic supplementary material

Supplementary information

Catalytic mechanism of amorphadiene synthase
