# Supplementary material for: Catalysis of amorpha-4,11-diene synthase unraveled and improved by mutability landscape guided engineering
Source: Sci Rep. 2018 Jul 2;8:9961. doi: 10.1038/s41598-018-28177-4 (PMC6028579; doi:10.1038/s41598-018-28177-4)
Supplement: Supplementary file 1 — Supplementary information [file 41598_2018_28177_MOESM1_ESM.pdf]

Catalysis of amorpha-4,11-diene synthase unraveled and improved by mutability landscape guided engineering

Ingy I. Abdallah, Ronald van Merkerk, Esmée Klumpenaar and Wim J. Quax*

Department of Chemical and Pharmaceutical Biology, Groningen Research Institute of Pharmacy, University of Groningen, 9713 AV, Groningen, The Netherlands.

*Corresponding author: Prof. Wim J. Quax, Department of Chemical and Pharmaceutical Biology, Groningen Research Institute of Pharmacy, University of Groningen, 9713 AV, Groningen, The Netherlands. Tel.: +31 (0) 50 363 2558, (0) 50 363 7660; fax: +31 (0) 50 363 3000.

E-mail address: w.j.quax@rug.nl

Supplementary information

Table S1: List of primers used to create the mutant library of ADS

**Residue**	**Primers**	
His 392	F1	CCAACCACTGAAGAGNDTGATCCAGTTGTAATC
	R1	GATTACAACTGGATCAHNCTCTTCAGTGGTTGG
	F2	CCAACCACTGAAGAGVMAGATCCAGTTGTAATC
	R2	GATTACAACTGGATCTKBCTCTTCAGTGGTTGG
	F3	CCAACCACTGAAGAGATGGATCCAGTTGTAATC
	R3	GATTACAACTGGATCCATCTCTTCAGTGGTTGG
	F4	CCAACCACTGAAGAGTGGGATCCAGTTGTAATC
	R4	GATTACAACTGGATCCCACTCTTCAGTGGTTGG
Val 396	F1	GAGCATGATCCAGTTNDTATCATTACTGGCGGTGCTA
	R1	TAGCACCGCCAGTAATGATAHNAACTGGATCATGCTC
	F2	GAGCATGATCCAGTTVMAATCATTACTGGCGGTGCTA
	R2	TAGCACCGCCAGTAATGATTKBAACTGGATCATGCTC
	F3	GAGCATGATCCAGTTATGATCATTACTGGCGGTGCTA
	R3	TAGCACCGCCAGTAATGATCATAACTGGATCATGCTC
	F4	GAGCATGATCCAGTTTGGATCATTACTGGCGGTGCTA
	R4	TAGCACCGCCAGTAATGATCCAAACTGGATCATGCTC
His 448	F1	TGATCTCATGACCNDTAAGGCCGAGCAAGAAAG
	R1	CTTTCTTGCTCGGCCTTAHNGGTCATGAGATCA
	F2	TGATCTCATGACCVMAAAGGCCGAGCAAGAAAG
	R2	CTTTCTTGCTCGGCCTTTKBGGTCATGAGATCA
	F3	TGATCTCATGACCATGAAGGCCGAGCAAGAAAG
	R3	CTTTCTTGCTCGGCCTTCATGGTCATGAGATCA
	F4	TGATCTCATGACCTGGAAGGCCGAGCAAGAAAG
	R4	CTTTCTTGCTCGGCCTTCCAGGTCATGAGATCA
Arg 440	F1	CTCAGGTATACTTGGTNDTCGCCTAAATGATCTC
	R1	GAGATCATTTAGGCGAHNACCAAGTATACCTGAG
	F2	CTCAGGTATACTTGGTVMACGCCTAAATGATCTC
	R2	GAGATCATTTAGGCGTKBACCAAGTATACCTGAG
	F3	CTCAGGTATACTTGGTATGCGCCTAAATGATCTC
	R3	GAGATCATTTAGGCGCATACCAAGTATACCTGAG
	F4	CTCAGGTATACTTGGTTGGCGCCTAAATGATCTC
	R4	GAGATCATTTAGGCGCCAACCAAGTATACCTGAG
Leu 515	F1	GATCTATTTGTGCCAGTTTNDTGAAGTTCAATATGC
	R1	GCATATTGAACTTCAHNAAACTGGCACAAATAGATC
	F2	GATCTATTTGTGCCAGTTTVMAGAAGTTCAATATGC
	R2	GCATATTGAACTTCTKBAAACTGGCACAAATAGATC
	F3	GATCTATTTGTGCCAGTTTATGGAAGTTCAATATGC
	R3	GCATATTGAACTTCCATAAACTGGCACAAATAGATC
	F4	GATCTATTTGTGCCAGTTTTGGGAAGTTCAATATGC
	R4	GCATATTGAACTTCCCAAAACTGGCACAAATAGATC

Trp 271	F1	GTTGAATGCTACTTTNDTGGACTAGGTTCAGGCTATG
	R1	CATAGCCTGAACCTAGTCCAHNAAAGTAGCATTCAAC
	F2	GTTGAATGCTACTTTVMAGGACTAGGTTCAGGCTATG
	R2	CATAGCCTGAACCTAGTCCTKBAAAGTAGCATTCAAC
	F3	GTTGAATGCTACTTTATGGGACTAGGTTCAGGCTATG
	R3	CATAGCCTGAACCTAGTCCCATAAAGTAGCATTCAAC
Arg 262	F1	GAACGCACCTTGTTTANDTGATAGAATTGTTGAATG
	R1	CATTCAACAATTCTATCAHNTAAACAAGGTGCGTTC
	F2	GAACGCACCTTGTTTAVMAGATAGAATTGTTGAATG
	R2	CATTCAACAATTCTATCTKBTAAACAAGGTGCGTTC
	F3	GAACGCACCTTGTTTAATGGATAGAATTGTTGAATG
	R3	CATTCAACAATTCTATCCATTAAACAAGGTGCGTTC
	F4	GAACGCACCTTGTTTATGGGATAGAATTGTTGAATG
	R4	CATTCAACAATTCTATCCCATAAACAAGGTGCGTTC
Phe 525	F1	GCAGGAAAGGATAACNDTACACGTATGGGAGAC
	R1	GTCTCCCATACGTGTAHNGTTATCCTTTCCTGC
	F2	GCAGGAAAGGATAACVMAACACGTATGGGAGAC
	R2	GTCTCCCATACGTGTTKBGTTATCCTTTCCTGC
	F3	GCAGGAAAGGATAACATGACACGTATGGGAGAC
	R3	GTCTCCCATACGTGTCATGTTATCCTTTCCTGC
	F4	GCAGGAAAGGATAACTGGACACGTATGGGAGAC
	R4	GTCTCCCATACGTGTCCAGTTATCCTTTCCTGC
Gln 518	F1	GCCAGTTTCTTGAAGTTNDTTATGCAGGAAAGG
	R1	CCTTTCCTGCATAAHNAACTTCAAGAAACTGGC
	F2	GCCAGTTTCTTGAAGTTVMATATGCAGGAAAGG
	R2	CCTTTCCTGCATATKBAACTTCAAGAAACTGGC
	F3	GCCAGTTTCTTGAAGTTATGTATGCAGGAAAGG
	R3	CCTTTCCTGCATACATAACTTCAAGAAACTGGC
	F4	GCCAGTTTCTTGAAGTTTGGTATGCAGGAAAGG
	R4	CCTTTCCTGCATACCAAACTTCAAGAAACTGGC
Lys 449	F1	GATCTCATGACCCACNDTGCCGAGCAAGAAAG
	R1	CTTTCTTGCTCGGCAHNGTGGGTCATGAGATC
	F2	GATCTCATGACCCACVMAGCCGAGCAAGAAAG
	R2	CTTTCTTGCTCGGCTKBGTGGGTCATGAGATC
	F3	GATCTCATGACCCACATGGCCGAGCAAGAAAG
	R3	CTTTCTTGCTCGGCCATGTGGGTCATGAGATC
	F4	GATCTCATGACCCACTGGGCCGAGCAAGAAAG
	R4	CTTTCTTGCTCGGCCCAGTGGGTCATGAGATC
Thr 296	F1	GCTGTTGCTGTTATANDTCTTATAGATGACACTTATG
	R1	CATAAGTGTCATCTATAAGAHNTATAACAGCAACAGC
	F2	GCTGTTGCTGTTATAVMACTTATAGATGACACTTATG
	R2	CATAAGTGTCATCTATAAGTKBTATAACAGCAACAGC
	F3	GCTGTTGCTGTTATAATGCTTATAGATGACACTTATG
	R3	CATAAGTGTCATCTATAAGCATTATAACAGCAACAGC

	F4	GCTGTTGCTGTTATATGGCTTATAGATGACACTTATG
	R4	CATAAGTGTCATCTATAAGCCATATAACAGCAACAGC
Tyr 519	F1	GCCAGTTTCTTGAAGTTCAANDTGCAGGAAAGG
	R1	CCTTTCCTGCAHNTTGAACTTCAAGAAACTGGC
	F2	GCCAGTTTCTTGAAGTTCAAVMAGCAGGAAAGG
	R2	CCTTTCCTGCTKBTTGAACTTCAAGAAACTGGC
	F3	GCCAGTTTCTTGAAGTTCAAATGGCAGGAAAGG
	R3	CCTTTCCTGCCATTTGAACTTCAAGAAACTGGC
	F4	GCCAGTTTCTTGAAGTTCAATGGGCAGGAAAGG
	R4	CCTTTCCTGCCCATTGAACTTCAAGAAACTGGC
Gly 400	F1	CCAGTTGTAATCATTACTNDTGGTGCTAACCTGC
	R1	GCAGGTTAGCACCAHNAGTAATGATTACAACTGG
	F2	CCAGTTGTAATCATTACTVMAGGTGCTAACCTGC
	R2	GCAGGTTAGCACCTKBAGTAATGATTACAACTGG
	F3	CCAGTTGTAATCATTACTATGGGTGCTAACCTGC
	R3	GCAGGTTAGCACCCATAGTAATGATTACAACTGG
	F4	CCAGTTGTAATCATTACTTGGGGTGCTAACCTGC
	R4	GCAGGTTAGCACCCCAAGTAATGATTACAACTGG
Gly 439	F1	GATACTCAGGTATACTTNDTCGACGCCTAAATGATCTC
	R1	GAGATCATTTAGGCGTCGAHNAAGTATACCTGAGTATC
	F2	GATACTCAGGTATACTTVMACGACGCCTAAATGATCTC
	R2	GAGATCATTTAGGCGTCGTKBAAGTATACCTGAGTATC
	F3	GATACTCAGGTATACTTATGCGACGCCTAAATGATCTC
	R3	GAGATCATTTAGGCGTCGCATAAGTATACCTGAGTATC
	F4	GATACTCAGGTATACTTTGGCGACGCCTAAATGATCTC
	R4	GAGATCATTTAGGCGTCGCCAAAGTATACCTGAGTATC
Asp 523	F1	CAATATGCAGGAAAGNDTAACTTCACACGTATGG
	R1	CCATACGTGTGAAGTTAHNCTTTCCTGCATATTG
	F2	CAATATGCAGGAAAGVMAAACTTCACACGTATGG
	R2	CCATACGTGTGAAGTTTKBCTTTCCTGCATATTG
	F3	CAATATGCAGGAAAGATGAACTTCACACGTATGG
	R3	CCATACGTGTGAAGTTCATCTTTCCTGCATATTG
	F4	CAATATGCAGGAAAGTGGAACTTCACACGTATGG
	R4	CCATACGTGTGAAGTTCCACTTTCCTGCATATTG
Thr 399	F1	CCAGTTGTAATCATTNDTGGCGGTGCTAACCTGC
	R1	GCAGGTTAGCACCGCCAHNAATGATTACAACTGG
	F2	CCAGTTGTAATCATTVMAGGCGGTGCTAACCTGC
	R2	GCAGGTTAGCACCGCCTKBAATGATTACAACTGG
	F3	CCAGTTGTAATCATTATGGGCGGTGCTAACCTGC
	R3	GCAGGTTAGCACCGCCCATAATGATTACAACTGG
	F4	CCAGTTGTAATCATTTGGGGCGGTGCTAACCTGC
	R4	GCAGGTTAGCACCGCCCCAAATGATTACAACTGG

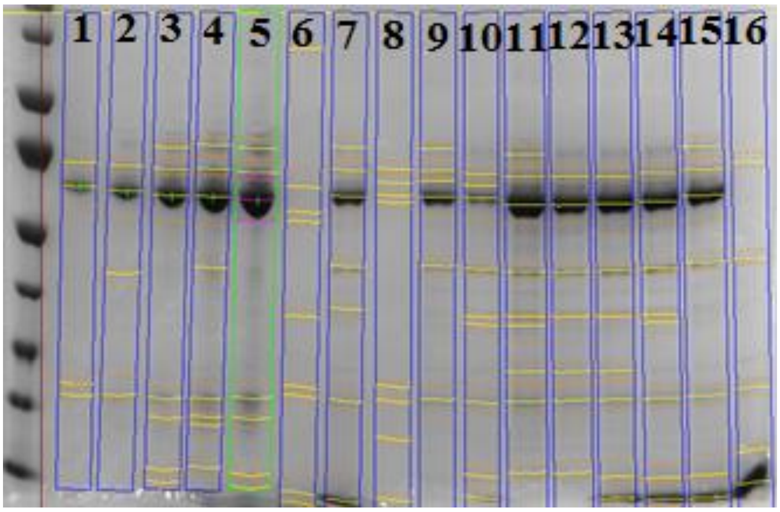

Figure S1. Digital image of a typical SDS-page gel used in the densitometric quantitation of ADS concentration. Lanes 1, 2, 3, 4 and 5 contain calibration samples of known concentrations of standard purified wild type ADS (resp. 100, 250, 500, 750 and 1000 ng/µl). The PageRulerTM prestained protein ladder (Thermo Scientific) was placed at the edge of the gel. Lanes 6-16 contain purified ADS, each representing a different variant. The blue boxes represent lanes and dashed orange lines indicate an area in which a protein band is detected by the program ‘GeneTools’.

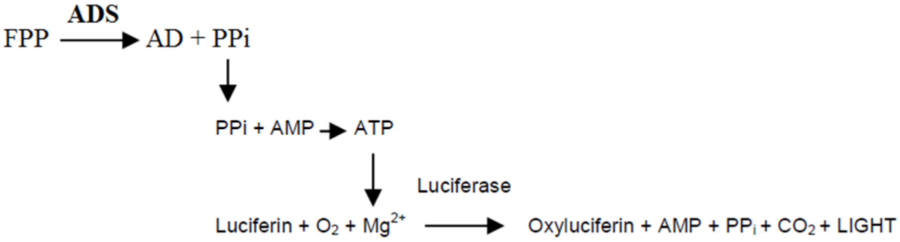

Figure S2. Theory of the bioluminescent assay for catalytic activity of ADS.

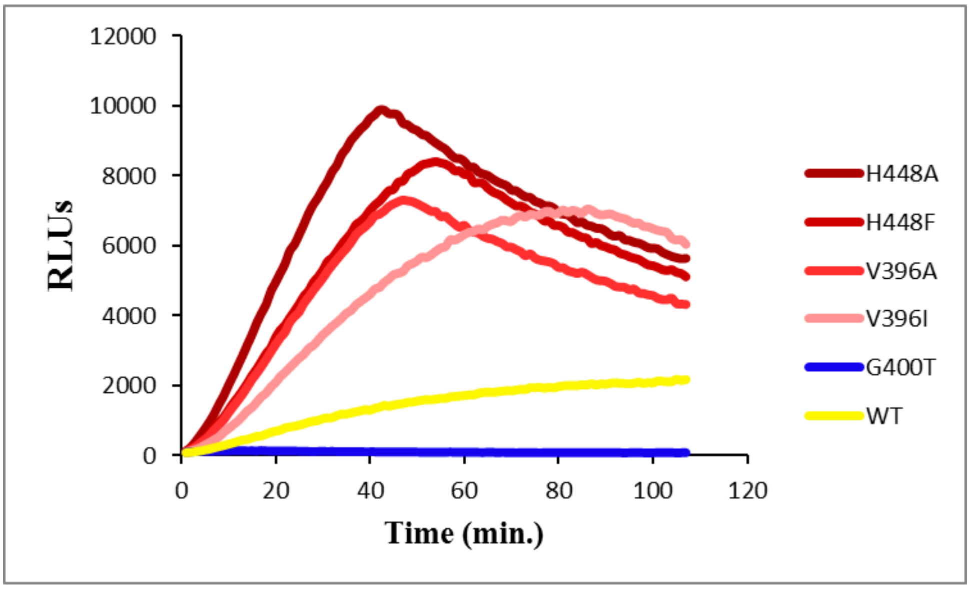

Figure S3. An example of the RLUs versus time curves produced by the bioluminescent assay. The curve of wild type ADS is presented in yellow. The variants with higher rate of reaction compared to wild type are shown in shades of red while inactive variants are depicted in blue. The slope of the linear part of these curves represents catalytic rate of reaction (V).

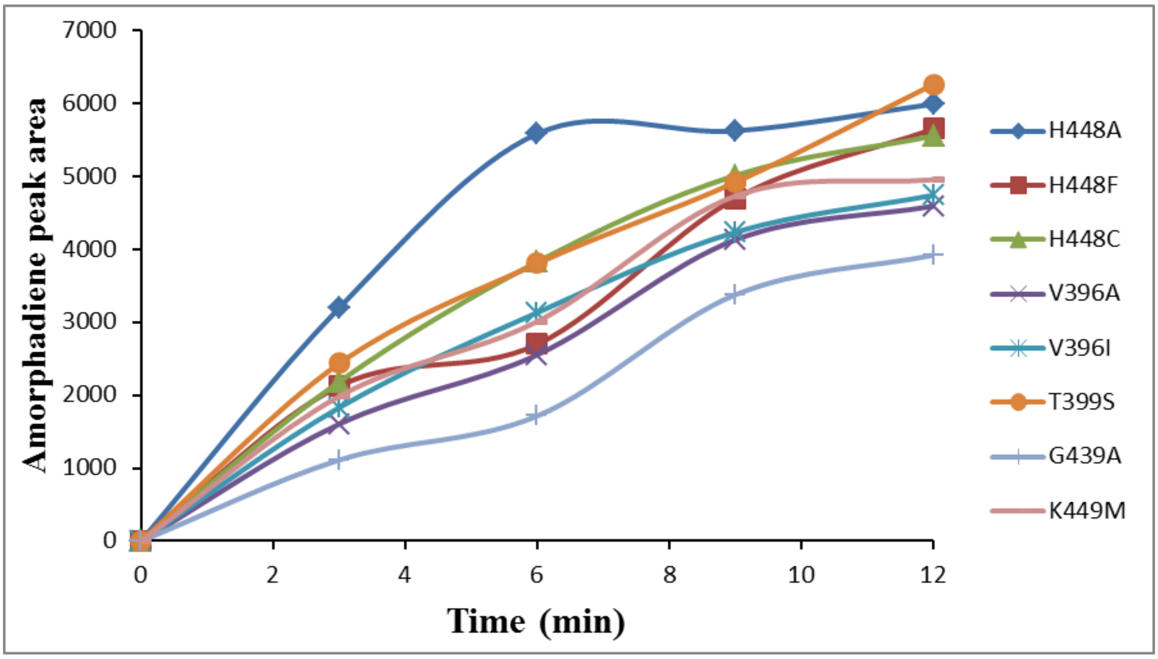

Figure S4. An example of the amorphadiene peak area versus time curves produced by the GC-MS assay. The slope of the linear part of these curves represents catalytic rate of reaction (V).

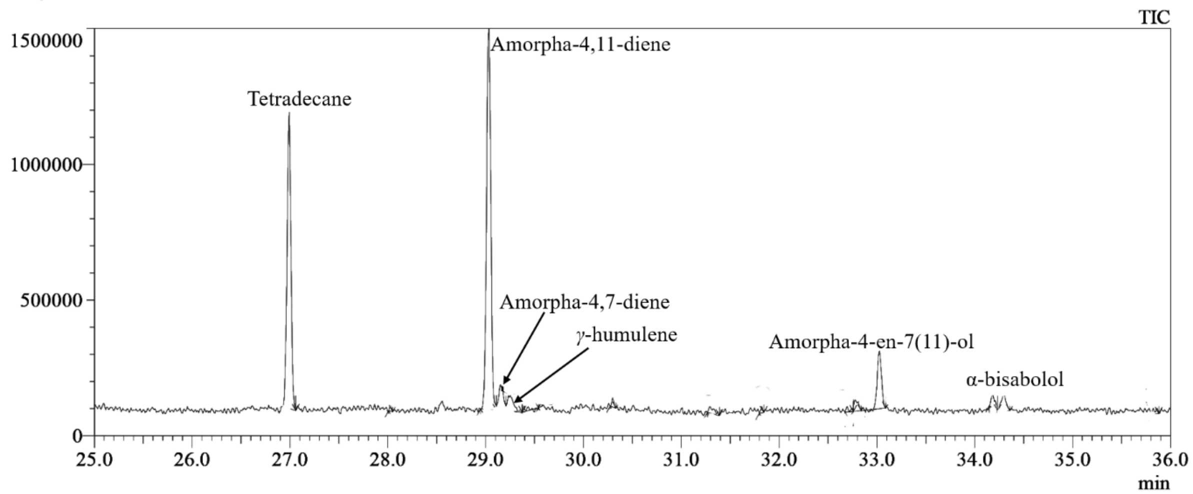

Figure S5. GC chromatogram of wild type ADS

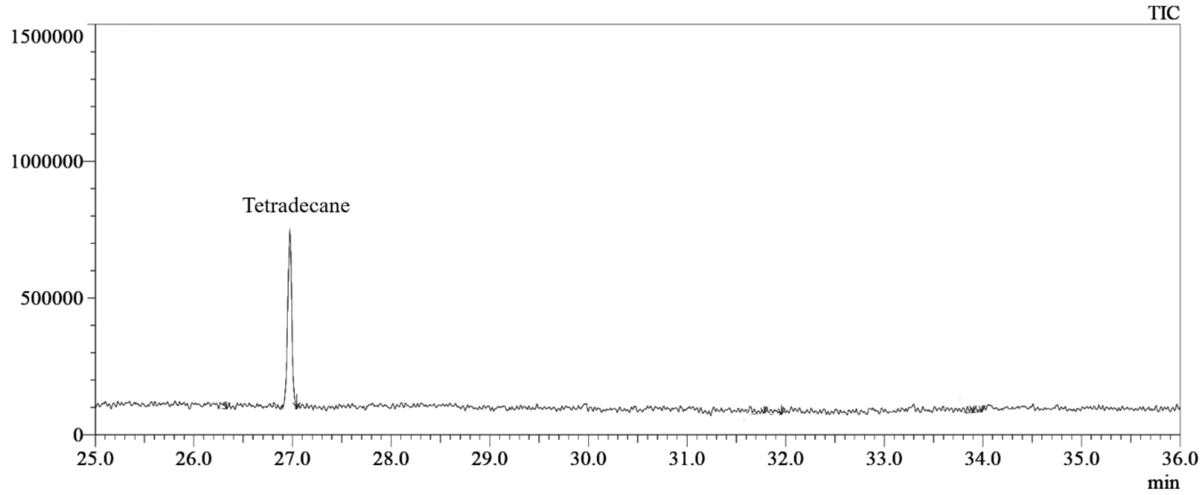

Figure S6. Example of GC chromatogram of inactive variants

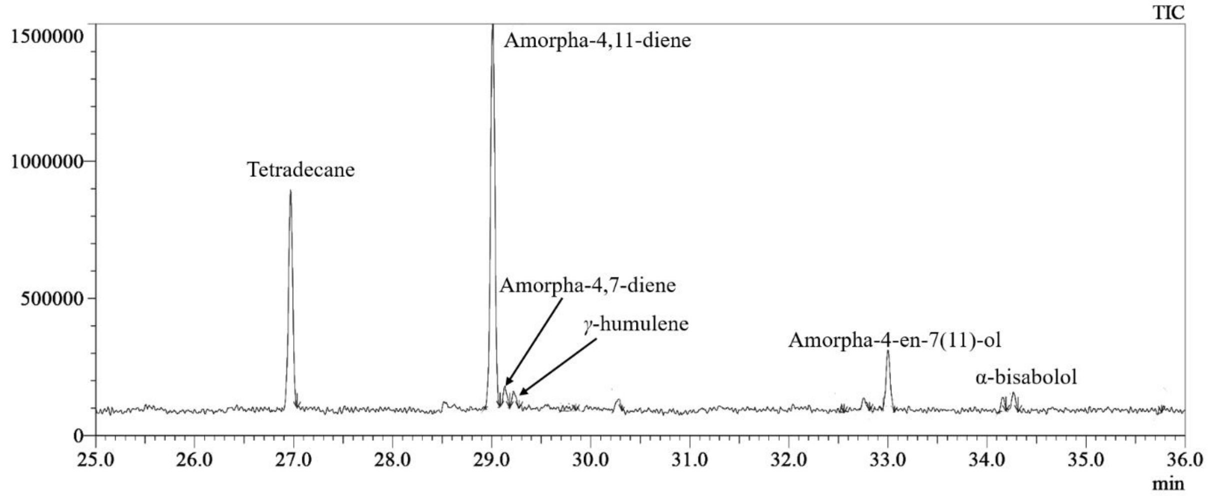

Figure S7. Example of GC chromatogram of variants showing the same product profile as that of the wild type ADS

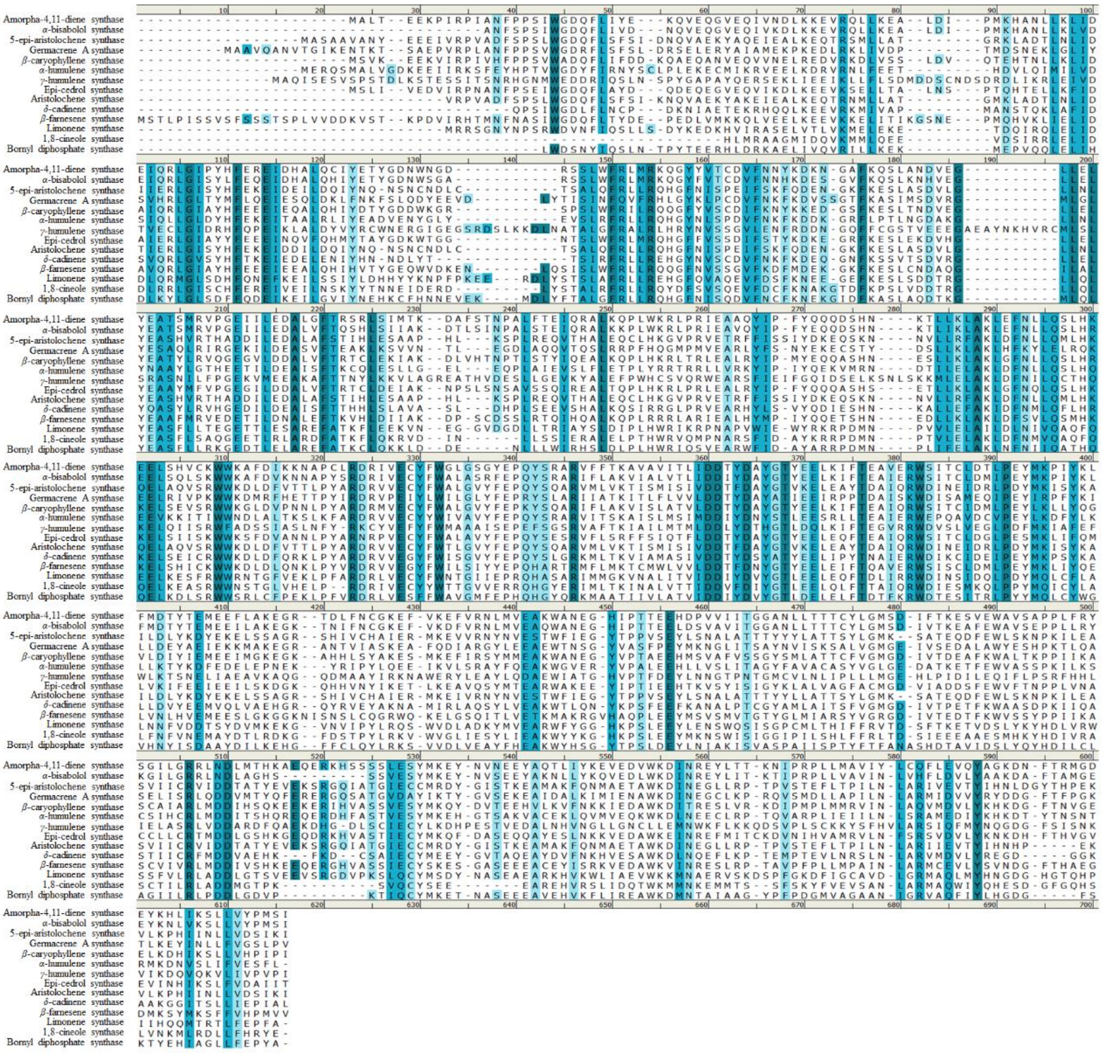

Figure S8. Sequence alignment of amorpha-4,11-diene synthase with ten sesquiterpene synthases and three monoterpene synthases
